# Marker-Assisted Improvement of Bread Wheat Variety HD2967 for Leaf and Stripe Rust Resistance

**DOI:** 10.3390/plants11091152

**Published:** 2022-04-24

**Authors:** Niharika Mallick, Shailendra K. Jha, Priyanka Agarwal, Anchal Mall, Niranjana M., Sachin Kumar, Manish K. Choudhary, Shreshtha Bansal, M. S. Saharan, J. B. Sharma, Vinod

**Affiliations:** 1 Division of Genetics, ICAR-Indian Agricultural Research Institute, New Delhi 110012, India; jhashail78@gmail.com (S.K.J.); priyankaagarwal11@gmail.com (P.A.); mall.anchal25@gmail.com (A.M.); mniranjana2010@gmail.com (N.M.); sachinkumar305222@gmail.com (S.K.); manishchoudhary6917@gmail.com (M.K.C.); shreshtha96@gmail.com (S.B.); jbsiari@gmail.com (J.B.S.); 2 Department of Genetics & Tree Propagation, Forest Research Institute, Dehradun 248006, India; 3 Division of Plant Pathology, ICAR-Indian Agricultural Research Institute, New Delhi 110012, India; mssaharan7@gmail.com

**Keywords:** marker-assisted selection, leaf rust, stripe rust, backcross breeding, near isogenic line, wheat

## Abstract

The mega wheat variety HD2967 was improved for leaf and stripe rust resistance by marker-assisted backcross breeding. After its release in 2011, HD2967 became susceptible to stripe rust and moderately susceptible to leaf rust. The leaf rust resistance gene *LrTrk* was transferred into HD2967 from the durum wheat genotype Trinakria. Then, HD2967 was crossed with Trinakria to produce F_1_ plant foreground selection for *LrTrk* and background selection for the recurrent parent genotype was carried out in BC_1_F_1_, BC_2_F_1_ and BC_2_F_2_ generations. Foreground selection was carried out with the linked marker *Xgwm234*, while polymorphic SSR markers between parents were used for background selection. Background selection resulted in the rapid recovery of the recurrent parent genome. A morphological evaluation of 6 near isogenic lines (NILs)—2 resistant to leaf and stripe rust, and 4 resistant to leaf rust only—showed no significant differences in yields among NILs and the recurrent parent HD2967. All of the 6 NILs showed the presence of 2NS/2AS translocation, carrying the linked genes *Lr37/Sr38/Yr17* present in HD2967 and the targeted leaf rust resistance gene *LrTrk*. Two NILs also showed additional resistance to stripe rust. Therefore, these NILs with rust resistance and an at par yielding ability of H2967 can replace the susceptible cultivar HD2967 to reduce yield losses due to disease.

## 1. Introduction

Bread wheat (*Triticum aestivum* L.) is one of the major food crops of the world, accounting for 20% of the calories consumed by humans globally [1]. The production of wheat is affected by several biotic and abiotic factors. Among the biotic factors, rust diseases caused by *Puccinia* spp. inflict significant damage to the crop of susceptible cultivars, resulting in substantial yield losses [2,3]. There are three rust diseases: viz. leaf rust (*Puccinia triticina* Eriks.); stem rust (*Puccinia graminis f.* sp. *Tritici*); and stripe rust (*Puccinia striiformis*), which infecting wheat under different agro-ecological conditions. Wheat growing areas are differentially suited to the development of leaf, stem, and stripe rust [4]. The leaf rust pathogen has a wide range of adaptation to different environments; and, it therefore occurs in all wheat growing areas, causing significant yield losses in susceptible cultivars globally [5,6,7]. Stripe rust is a devastating disease affecting wheat worldwide, especially under cool and moist conditions [8,9]. Stem rust develops under relatively warmer conditions [10,11] and it can cause substantial yield losses, especially under epidemic conditions [12,13,14]. In India, among all the three rusts, leaf rust is the most widespread and prevails in all the wheat-growing zones, while stripe rust occurs predominantly in the cooler areas of the north–western plains and northern hill states in the Himalayas. Stem rust is a disease of warmer areas and occurs mainly in central and peninsular India. Although fungicides can control rust diseases, developing rust-resistant cultivars is an environment friendly and economical method of disease control [15,16]. The evolution of new and virulent pathotypes of the rust pathogen renders the existing cultivars susceptible. There is a need for the identification of new and effective sources of resistance and their utilization in breeding programs [17,18,19]. There is a continuous need for developing new cultivars with effective resistance genes to replace the susceptible cultivars. With developments in molecular genetics and genomics, marker-assisted breeding has emerged as a major tool in varietal development. Marker-assisted backcross breeding provides a precise method to transfer rust resistant genes in an agronomically well-adapted cultivar that has become susceptible due to the evolution of new virulent pathotypes [20,21,22,23]. Two wheat varieties—Unnat PBW343 and Unnat PBW550—were developed using marker-assisted backcross breeding [24,25] and they were released for cultivation in 2017 to replace the popular but susceptible wheat varieties of India PBW343 and PBW550, respectively. These varieties are giving higher returns to farmers [26].

Developed at the Indian Agricultural Research Institute in New Delhi, wheat variety ICAR-HD2967 is a mega variety released for general cultivation in India’s North Western Plain Zone under timely sown irrigated conditions in 2011 [27]. Variety HD2967 soon became popular and occupied more than 10 million hectares [28]. Due to its adaptability and its high yield, HD2967 was also recommended for cultivation in the North Eastern Plain Zone. However, over a period of time, HD2967 became susceptible to stripe rust and it showed moderate susceptibility to leaf rust. Both stripe rust and leaf rust are important diseases of the NWPZ (North Western Plain Zone) as well as the NEPZ (North Eastern Plain Zone) due to conducive environmental conditions during the crop season. Due to its yield advantage and its adaptability, HD2967 remains popular among farmers; therefore, the transfer of leaf and stripe rust resistance genes to HD2967 can protect farmers from yield losses and reduce spending on fungicides. Durum wheat genotype Trinakria showed leaf and stem rust resistance under field conditions [29]. Trinakria also showed a high degree of stripe rust resistance at the seedling and the adult plant stages. In the present study, Trinakria was used as a donor for leaf and stripe rust resistance in an effort to improve leaf and stripe rust resistance in HD2967.

## 2. Results

### 2.1. Development of NILs Carrying Leaf Rust Resistance Gene LrTrk and Leaf and Stripe Rust Resistance Gene LrTrk/YrTrk

**Figure 1:**
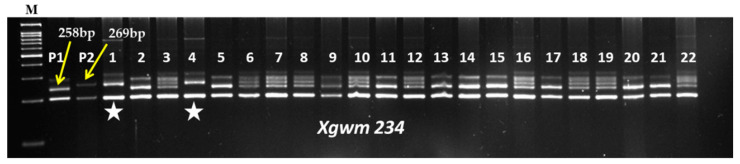
Representative gel picture of foreground selection for *LrTrk* in BC_2_F_2_ generation. Here, M: 100 bp ladder; P1: HD2967; P2: Trinakria; 1–22: BC_2_F_2_ plants; 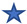: Plants homozygous for *LrTrk*.

Crosses were made between the recurrent parent (RP) HD2967 and the donor parent (DP) Trinakria (Tetraploid donor) to produce the F_1_ generation. The co-dominant SSR marker *Xgwm234* was linked with the leaf rust resistance gene *LrTrk* to confirm the heterozygosity of F_1_ plants. Five true F_1_ plants were backcrossed with HD2967 to produce the BC_1_F_1_ generation. The BC_1_F_1_ seeds were found to be a mixture of normal-filled and shriveled seeds. A total of 145 normal-filled BC_1_F_1_ seeds were sown; out of that, 60 plants were found to carry *LrTrk* in the heterozygous state when screened with the *Xgwm234* marker (Table 1). Out of the 60 BC_1_F_1_ plants, 10 plants that looked phenotypically similar to HD2967 were selected for marker-assisted background analysis. A parental polymorphism survey between HD2967 and Trinakria with 700 SSR markers (Table A1) identified 83 polymorphic markers (Table A2). A background analysis with polymorphic SSR markers of 10 phenotypically selected BC_1_F_1_ plants showed that RPG recovery varied from 78.91% to 83.13% (Table 1). The plant carrying maximum RPG recovery of 83.13% was backcrossed with HD2967 to produce the BC_2_F_1_ generation. As compared to the BC_1_F_1_ generation, BC_2_F_1_ seeds were found to be normal and well-filled. A total of 66 BC_2_F_1_ plants were screened for the leaf rust resistance gene *LrTrk* with the linked SSR marker *Xgwm234*. Thirty-nine plants were identified as carrying *LrTrk* in heterozygous conditions (Table 1). Again, ten plants that looked phenotypically similar to HD2967 were selected for background analysis using SSR markers. In the ten selected plants in the BC_2_F_1_ generation, RPG recovery varied from 90.36% to 93.37% (Table 1). The plant with a maximum RPG recovery of 93.37% was selfed to produce the BC_2_F_2_ generation. Foreground selection among 200 BC_2_F_2_ plants was undertaken that identified 98 and 61 plants carrying the leaf rust resistance gene *LrTrk* (a 269 bp band) in heterozygous and homozygous states, respectively (Figure 1; Table 1). A background analysis revealed that RPG recovery in 61 BC_2_F_2_ plants homozygous for *LrTrk* ranged from 95.18% to 98.79%. Thirty-two homozygous BC_2_F_2_ plants with RPG recovery above 97% were selfed to produce BC_2_F_3_ families (Table 1).

**Figure 2:**
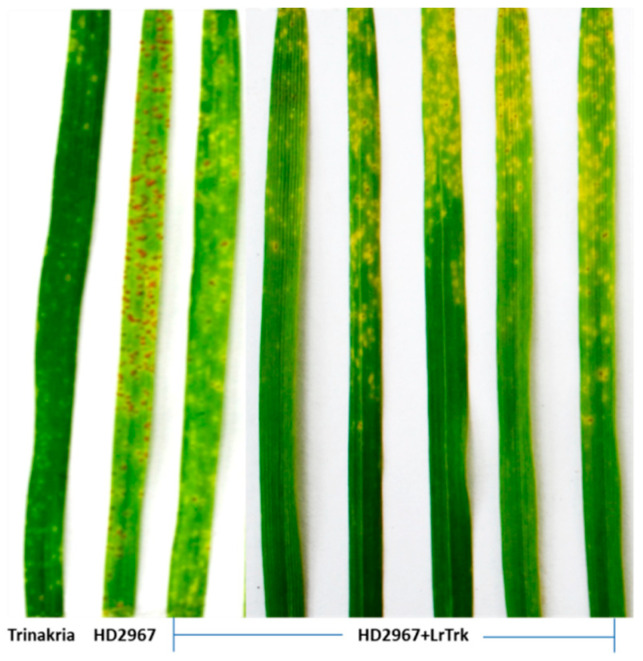
NILs (HD2967 + *LrTrk*) along with their parents, HD2967, and Trinakria showing seedling response to leaf rust pathotype 77-5.

**Figure 3:**
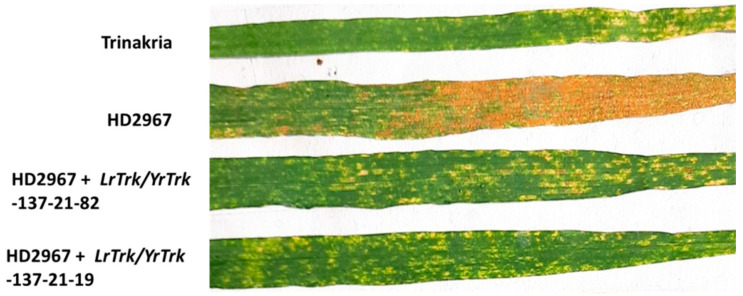
HD2967 + *LrTrk/YrTrk* NILs along with their parents HD2967 and Trinakria showing adult plant response to stripe rust race 110S119.

Thirty-two BC_2_F_3_ NILs and their RP HD2967 and DP Trinakria were also screened for leaf and stripe rust resistance at the seedling and the adult plant stages, respectively. Out of 32 NILs, 30 NILs were found to be resistant to leaf rust with I.T. ‘;1’ (Figure 2, Table 2 and Table 3), while 2 NILs gave a susceptible reaction with I.T. ‘3’. Of the 32 NILs tested for stripe rust resistance at the adult plant stage, 14 NILs were resistant. These 14 NILs were also resistant to leaf rust (Table 2). Thus, of the 32 NILs screened for rust resistance, 16 NILs were found to be resistant to leaf rust only, while 14 NILs showed resistance to both leaf and stripe rusts (Table 2). The NILs with only the leaf rust resistance gene *LrTrk* are henceforth referred to as HD2967 + *LrTrk,* while those with leaf and stripe rust resistance are referred to as HD2967 + *LrTrk*/*YrTrk* (*YrTrk* for stripe rust resistance gene(s) in Trinakria) in this paper. When tested against the leaf rust pathotype 77-5, HD2967 showed susceptibility to leaf rust at the seedling stage with an I.T. of ‘3^-^’ (Figure 2). When screened against the stripe rust pathotype 110S119 at the adult plant stage (rust response 60S), HD2967 was susceptible to stripe rust (Figure 3). Trinakria, the durum wheat genotype used as a donor for leaf and stripe rust resistance, displayed a high degree of leaf rust resistance with an I.T. ‘;’ (Figure 2; Table 3) and a resistance response (10R) against the stripe rust pathotype 110S119 at the adult plant stage (Figure 3; Table 3). The response of NILs to the leaf rust pathotype 77-5 and the stripe rust pathotype 110S119 can be seen in Figure 2 and Figure 3, respectively. All of the 6 NILs showed I.T. ‘;1′ to the leaf rust pathotype 77-5 when tested at the seedling stage, whereas only 2 NILs (HD2967 + *LrTrk/YrTrk*-137-21-82, HD2967 + *LrTrk/YrTrk*-137-21-19) showed a resistance response (10R) toward the stripe rust pathotype 110S119 at the adult plant stage (Figure 3).

**Figure 4:**
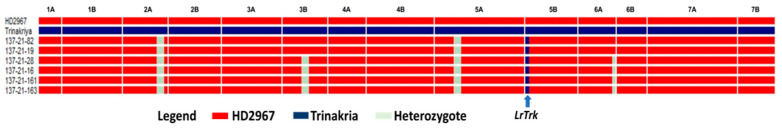
Graphical representation of HD2967 NILs carrying leaf rust resistance gene *LrTrk* and leaf and stripe rust resistance gene *LrTrk/YrTrk*, showing the extent of recurrent parent genome recovery.

**Figure 5:**
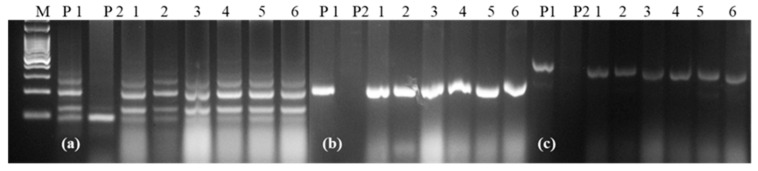
Representative gel picture showing recovery of the D genome in all of the NILs derived from HD2967; (**a**) *Xcfd67*, (**b**) *Xcfd84*, (**c**) *Xcfd165*: D genome specific markers; M: 100 bp ladder, P1: HD2967, P2: Trinakria, 1–6: HD2967 NILs carrying *LrTrk*.

**Figure 6:**
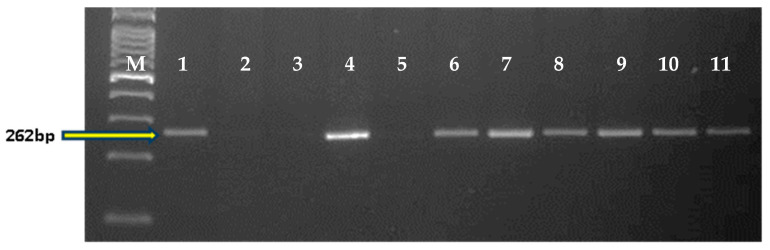
Amplification with 2NS specific primer pair, VENTRIUP, and LN2. Here, M: 100 bp ladder, 1: Thatcher+*Lr37* (+ve control); 2: Agra Local; 3: Kharchia Local; 4: HD2967; 5: Trinkria; 6–11: HD2967 + *LrTrk* NILs.

Based on the yield, seed selection, and rust score of BC_2_F_3_ families, 6 NILs were selected for a detailed evaluation in replicated yield trials. Two of the selected NILs were resistant to leaf and stripe rusts (Table 3), while the remaining four showed resistance to leaf rust only. The RPG recovery of these 6 NILs ranged from 97.59% to 98.79%. Graphical representation of the 6 NILs showed recovery of the recurrent parent genome in all chromosomes except in chromosomes 2A, 3B, 5A and 6A, where some residual donor segments were found to be present in the heterozygous state (Figure 4). For the background analysis of the 6 NILs in the BC_2_F_4_ generation, D genome-specific SSR markers were also used. It was observed that all of the D genome-specific markers were monomorphic between HD2967 and HD2967 + *LrTrk/YrTrk* NILs, and no amplification was observed in Trinakria (Figure 5). Marker analysis with 2NS/2AS specific markers showed that all of these six NILs carried *Ae. ventricosa* translocation, having rust resistance genes *Lr37/Sr38/Yr17* (Figure 6).

### 2.2. Evaluation of HD2967 NILs for Yield-Related Traits

Six NILs were selected for a detailed evaluation of agro-morphological traits in replicated trials based on their yield in the BC_2_F_3_ generation, seed selection, and rust evaluation. These 6 NILs consisted of 4 NILs with only leaf rust resistance and 2 NILs with leaf and stripe rust resistance. The mean performance of six near isogenic lines for yield and yield-related traits is presented in Table 4. While all of the NILs were found to have similar heights as that of RP HD2967, the NIL HD2967 + *LrTrk/YrTrk*-137-21-82 was observed to be significantly taller. The NILs HD2967 + *LrTrk/YrTrk*-137-21-82 and HD2967 + *LrTrk*-137-21-163 showed significant superiority for spike length (S.L.) compared to HD2967. Out of these two, NIL HD2967 + *LrTrk/YrTrk*-137-21-82 showed a significantly higher number of spikelets/spike (NSpl) than HD2967. The NIL HD2967 + *LrTrk*-137-21-16 showed a significantly lower spike length (S.L.) and a significantly lower number of spikelets/spike (NSpl). Though there was a difference in spike length (S.L.) and in the number of spikelets/spike (NSpl) in different NILs, all of the NILs showed at par performance for the trait number of seeds/spike (NS). Two NILs, HD2967 + *LrTrk*-137-21-28 and HD2967 + *LrTrk*-137-21-161, showed a significantly higher thousand kernel weight (TKW), but their yields were at par with RP HD2967. Overall, all of the NILs of HD2967 produced yield at par with HD2967, and the differences in yield were non-significant.

## 3. Discussion

The durum wheat genotype Trinakria showed a high degree of resistance against leaf and stripe rusts. A leaf rust resistant gene, tentatively named *LrTrk*, was mapped on chromosome 5BS in Trinakria [30]. The variety HD2967 is a popular bread wheat, and incorporation of leaf and stripe rust resistance from Trinakria will enhance the usefulness of the variety, which over the years has become highly susceptible to stripe rust, with a moderate susceptibility to leaf rust. Since the leaf rust resistance gene *LrTrk* in Trinakria was mapped, and the SSR marker *Xgwm234* was linked to the resistant gene, a marker-assisted backcrossing program was initiated to transfer the leaf rust resistance gene *LrTrk* into HD2967. Though information about a linkage between *LrTrk* and the stripe rust resistance gene(s) in Trinakria was not available, we presumed that some of the lines developed by selecting marker-assisted *LrTrk* would also be resistant to stripe rust, enabling us to choose lines carrying both leaf and stripe rust resistance in the genetic background of HD2967.

Trinakria is a durum wheat genotype and tetraploid wheat (2*n* = 4*x* = 28, genome AABB), while cultivar HD2967 is a hexaploid bread wheat (2*n* = 6*x* = 42, genome AABBDD). HD2967 was used as a female parent, and Trinakria was used as the pollen parent. All F_1_ plants are expected to be aneuploid (pentaploid) with 2*n* = 2*x* = 35 chromosomes and to show high pollen sterility. However, F_1_ plants can be easily emasculated and be crossed as a female parent with normal fertile pollens provided by the recurrent parent HD2967 in backcrossing. The spikes of five F_1_ plants were pollinated with HD2967 pollens to produce sufficient seeds for the BC_1_F_1_ generation. The BC_1_F_1_ seeds were a mixture of well-filled and shriveled seeds. This was on expected lines as F_1_ plants, being pentaploid, produce gametes with aneuploid chromosome numbers. The seven D genome chromosomes in F_1_ plants contributed by HD2967 segregate randomly during gamete formation. Theoretically, the chromosome number in gametes produced by F_1_ plants are expected to vary from 14 to 21. BC_1_F_1_ plants are expected to carry chromosome numbers ranging from 35 to 42. The BC_1_F_1_ seeds carrying unbalanced chromosome numbers are expected to have poor endosperm development, which was reflected in the BC_1_F_1_ seed, a mixture of seeds with poorly filled and well-filled endosperm. Only seeds with well-developed endosperm were sown. In the BC_1_F_I_ generation, though 60 plants were identified as carrying the leaf rust resistance gene *LrTrk*, only 10 plants resembling HD2967 phenotypically were selected for background selection. A plant with a maximum RPG recovery of 83.13% was chosen for further backcrossing. Phenotypic selection combined with marker-assisted background selection in the BC_1_F_1_, BC_2_F_1_ and BC_2_F_2_ generations resulted in a rapid recovery of the background genome of HD2967 from 83.13% in BC_1_F_1_ to 93.37% and 98.79% in the BC_2_F_1_ and the BC_2_F_2_ generations, respectively. However, the RPG recovery of 97.59–98.79% applies only to A and B genomes of NILs; the D genome in NILs is entirely derived from HD2967 and it is expected to remain unaltered. Molecular markers have been effectively used to select rust resistant genes in wheat [20,21,22,23]. The effectiveness of molecular markers is also reflected in our study wherein out of 32 NILs identified as carrying *LrTrk* with the linked marker *Xgwm234*, only two NILs were susceptible to leaf rust. At the same time, the remaining 30 lines were resistant. Crossing over between a molecular marker and a rust resistant gene is expected as *Xgwm234* is not a gene-specific marker. Screening of 32 NILs for stripe rust resistance in BC_2_F_3_ at the adult plant stage identified 14 NILs that carried stripe rust resistance. Thus, combining marker-assisted selection for leaf rust resistance and phenotypic selection for stripe rust resistance enabled the accelerated development of the NILs of the wheat variety HD2967 carrying resistance to both leaf and stripe rusts. Marker-assisted background selection accelerated the recovery of RPG of HD2967 with NILs in BC_2_F_3_ showing more than a 97% recovery of RPG. The marker-assisted background analysis was restricted to wheat’s A and B genome only because the donor parent Trinakria lacked the D genome. Thus, the entire D genome in NILs is expected from HD2967, which was also demonstrated in a polymorphism study among HD2967, Trinakria, and NILs. All of the D genome-specific markers used in the study were monomorphic between HD2967 and the NILs, and they failed to amplify in the donor parent Trinakria (Figure 5). The use of a tetraploid donor thus enabled the complete recovery of the D genome in the NILs of HD2967.

The wheat variety HD2967 was shown to carry *Ae. ventricosa* translocation 2NS/2AS, which harbors the linked APR genes *Lr37*, *Yr17* and *Sr38* [31]. Six NILs that were finally selected for yield evaluation were screened for 2NS specific markers. The results showed that all of the six NILs carried 2NS/2AS translocation. Thus, out of six NILs, four had 2NS/2AS translocation in addition to the *LrTrk* gene for leaf rust resistance, while the remaining two NILs carried *LrTrk/YrTrk* and 2NS/2AS translocation. While *Lr37* is an adult plant resistant gene, *LrTrk* is a seedling resistance gene. Additionally, *Lr37* is susceptible to several pathotypes of *P. triticina* [32]. Thus, *LrTrk* and *Lr37* will provide enhanced resistance against *P. triticina* in the NILs. Among the six NILs, two were resistant to both leaf and stripe rusts (Table 3). These two lines carried *YrTrk* along with *Yr17*; although *Yr17* is ineffective against *P. striiformis* pathotypes [33], making HD2967 susceptible to stripe rust. The NILs of HD2967 developed in this study will provide improved versions of HD2967 with leaf and stripe rust resistance and they will yield at par with HD2967.

## 4. Materials and Method

### 4.1. Plant Materials and Backcross Breeding Scheme

The bread wheat variety HD2967 was used as a recurrent parent in the backcross breeding program. The durum wheat genotype Trinakria was used as a donor for leaf and stripe rust resistance. Earlier, a leaf rust resistant gene named *LrTrk* was identified and mapped on chromosome 5BS in Trinakria [30]. Marker-assisted backcross breeding was used to transfer leaf rust resistance from Trinakria into HD2967 using a linked SSR marker, while conventional pathotype based screening was performed to select plants for stripe rust resistance. The variety HD2967 was crossed as a female parent with Trinakria to produce the F_1_ generation. The F_1_ generation was raised, and the hybridity of F_1_ plants was confirmed using the SSR marker *Xgwm234* (F: 5′ GAGTCCTGATGTGAAGCTGTTG 3′; R: 5′ CTCATTGGGGTGTGTACGTG 3′) linked to the leaf rust resistance gene *LrTrk*. True F_1_ plants were backcrossed with the recurrent parent (RP) HD2967 to produce the BC_1_F_1_ generation. Foreground selection was carried out for the leaf rust resistance gene *LrTrk* with the linked SSR marker *Xgwm234* in BC_1_F_1_. Plants carrying *LrTrk* were subsequently subjected to phenotypic selection for their resemblance to RP HD2967 before background selection using SSR markers showing polymorphism between HD2967 and Trinakria. Ten plants phenotypically resembling HD2967 were used for background analysis. A parental polymorphism survey between HD2967 and Trinakria was carried out with 700 SSR markers, well distributed across A and B genomes of wheat. The plant showing a maximum recovery of the recurrent parent genome (RPG) in the BC_1_F_1_ generation was again backcrossed to HD2967 to produce the BC_2_F_1_ generation. In the BC_2_F_1_ generation, foreground and background selections were also performed, as was done in the BC_1_F_1_ generation. The plant carrying *LrTrk* and a maximum RPG recovery was selfed to produce the BC_2_F_2_ generation. In the BC_2_F_2_ generation, plants having the leaf rust resistance gene *LrTrk* in the homozygous state were identified and analyzed for their background recovery. A plant with a maximum RPG recovery in the BC_2_F_2_ generation was self-pollinated by covering the spikes with butter paper bags to produce the BC_2_F_3_ families. The selection among the BC_2_F_3_ families was made based on the yield and the RPG%. The selected BC_2_F_4_ lines were evaluated in replicated yield trials.

### 4.2. Marker Analysis

DNA was extracted from one month old seedlings using the CTAB method [34]. The DNA samples were quantified, and their quality was confirmed using a NanoDrop^TM^ spectrophotometer. The DNA samples were diluted to a concentration of 25 ng/μL as working stock and then stored at −20 °C. A PCR reaction was carried out with SSRs in a reaction volume of 10 μL, comprising 4 μL of 2× GoTaq PCR Master Mix (Promega, #M7122), 1 μL of each primer (5 pmol/ul), 2 μL of nuclease-free water, and 2 μL of 25 ng/μL gDNA (50 ng) in 96-well PCR plates with a thermal seal in an Eppendorf thermal cycler. A thermal profile of 4 min at 94 °C (initial denaturation), followed by 35 cycles of 30 s at 94 °C (denaturation), 30 s at 50–60 °C (varying according to primer annealing temperature), and 30 s at 72 °C (primer extension), with a final extension at 72 °C for 10 min were used in a PCR machine for amplification of the SSR markers. The amplified products were resolved on 3.5% agarose gel and then visualized on a U.V. trans-illuminator Gel Documentation System (G: Box, Syngene). The RPG recovery was calculated as the number of homozygous loci corresponding to the recurrent parent + half the number of heterozygous loci/total number of polymorphic SSR markers used ×100. As parental polymorphism was not conducted for markers belonging to the D genome, a confirmation PCR was performed in the BC_2_F_4_ generation to identify the recovery of the D genome. Markers specific to the D genome were selected and then used for amplification in HD2967, Trinakria, and the six NILs carrying the *LrTrk* gene. The RPG recovery of 14 chromosomes belonging to the A and the B genomes of wheat was visualized using Graphical GenoTypes (GGT) Version 2.0 software [35].

The selected NILs were also screened for the presence of *Ae. ventricosa* translocation 2NS/2AS carrying linked rust resistance genes *Lr37*, *Yr17* and *Sr38* present in RP HD2967 using 2NS specific primer pair, VENTRIUP + LN2 [33]. The PCR reaction was performed according to the profile used by [36]. A Thatcher+*Lr37* (RL6081) was used as a positive control, whereas Agra Local and Kharchia Local were used as a negative control to confirm the presence of the 2NS/2AS translocation.

### 4.3. Screening of NILs for Rust Resistance

The NILs in the BC_2_F_3_ generation were screened for both leaf and stripe rust resistance. Screening for leaf rust resistance was carried out with the *P. triticina* pathotype 77-5 at the seedling stage in a glasshouse. Screening for stripe rust resistance was performed in the field with the *P. striiformis* pathotype 110S119 at the adult plant stage. In India, pathotypes 77-5 and 110S119 are some of the most virulent and prevalent pathotypes of leaf and stripe rusts, respectively. Initial inoculums were obtained from the ICAR-Indian Institute of Wheat and Barley Research (IIWBR), Regional Station, Flowerdale, Shimla, and they multiplied on the susceptible common wheat cultivar Agra Local at IARI, New Delhi.

For screening of leaf rust resistance, the NILs, RP HD2967, and susceptible check Agra Local were sown in aluminum trays (4 × 10 × 3 inches) in the glasshouse. Ten-day-old seedlings were inoculated with the leaf rust pathotype 77-5 by spraying the inoculum with a hand sprayer. The inoculation mixture was prepared by adding urediospores in water with a drop of Tween 20. After inoculation, the trays were kept in humid glass chambers for 48 h and subsequently shifted to glass house benches under ambient light and temperature conditions. A rust response (infection type) was recorded 12 days after inoculation, as described by Stakmann et al. (1962) [37].

For stripe rust screening, parents HD2967 and Trinakria and NILs carrying leaf rust resistance gene *LrTrk* were sown in yellow rust nursery in 1m rows each. Infector rows were planted after every 20 rows. To ensure uniform disease spread, one row of infector between two 1m row beds and two rows of infectors surrounding the test material were also planted. The spores of the stripe rust pathotype 110S119 were sprayed as a suspension in water fortified with Tween 20 at the booting stage. The inoculum mixture was sprayed thrice at the booting stage with two–three days interval. The plant response to stripe rust was scored based on the Modified Cobb’s scale [38] and disease severity (0–100%).

### 4.4. Evaluation of HD2967 + LrTrk NILs for Agro-Morphological Traits

Following the recommended package of practices at IARI, New Delhi, NILs, HD2967 + *LrTrk*, HD2967 + *LrTrk/YrTrk*, and the recurrent parent HD2967 were evaluated for agro-morphological traits in a randomized complete block design with two replications. The data on plant height (P.H.), spike length (S.L.), thousand kernel weight (TKW), the number of spikelets per spike (NSplSp), and the number of seeds per spike (NSSp) were recorded on 5 randomly selected plants from the inside rows of each plot. Each plot of 6 m^2^ size was harvested by machine and their plot yield (in kg) from each replication was recorded. The data on morphological traits was analyzed using OPSTAT statistical software (CCS HAU, Hisar) [39].

## Figures and Tables

**Table 1:** Number of gene-positive plants identified in each backcross generation and their background recovery.

Recipient Parent	Target Gene	Generation	No. of Plants Screened with Linked Molecular Marker	No. of Plants Carrying Target Gene		No. of Plants Selected for Background Selection	Number of Plants Backcrossed/Selfed/Selected	Recurrent Parent Genome (RPG) Recovery (%)
				Heterozygous	Homozygous			
HD2967	*LrTrk*	BC_1_F_1_	145	60	-	10	1	78.91–83.13
		BC_2_F_1_	66	39	-	10	1	90.36–93.37
		BC_2_F_2_	200	98	61	61	32	95.18–98.79

**Table 2:** Number of plants identified with leaf rust and stripe rust resistance in the BC_2_F_3_ generation and genome recovery of selected plants.

No. of plants screened for leaf and stripe rust	32
No. of plants resistant to leaf rust only	16
No. of plants resistant to both leaf and stripe rust	14
No. of plants selected for replicated trials	6
(RPG) recovery (%) of selected plants in replicated trial	97.59–98.79

**Table 3:** Phenotyping of NILs for leaf and stripe rust resistance at the seedling and the adult plant stages, respectively.

S. No.	Near Isogenic Lines of HD2967	ITs for Leaf Rust Race 77-5	Response to Stripe Rust Race 110S119
1	HD2967 + *LrTrk/YrTrk*-137-21-82	;1	10R
2	HD2967 + *LrTrk/YrTrk*-137-21-19	;1	10R
3	HD2967 + *LrTrk*-137-21-28	;1	40S
4	HD2967 + *LrTrk*-137-21-16	;1	30S
5	HD2967 + *LrTrk*-137-21-161	;1	40S
6	HD2967 + *LrTrk*-137-21-163	;1	40S
7	HD2967	3	60S
8	Trinakria	;	10R

**Table 4:** Morphological characterization of NILs of HD2967 carrying leaf rust resistance gene *LrTrk*.

NILs	PH	SL	NSpl	NS	TKW (gm)	YLD (kg)
HD2967 + *LrTrk/YrTrk*-137-21-82	111.40 *	13.72 *	25.80 *	75.60	37.25	3.94
HD2967 + *LrTrk/YrTrk*-137-21-19	104.40	12.62	23.40	73.20	37.75	3.69
HD2967 + *LrTrk*-137-21-28	100.20	11.04	22.20	71.40	42.00 *	4.02
HD2967 + *LrTrk*-137-21-16	103.20	10.52 *	21.80 *	68.20	37.00	3.42
HD2967 + *LrTrk*-137-21-161	99.80	11.68	22.60	71.80	42.00 *	4.11
HD2967 + *LrTrk* -137-21-163	101.40	13.18 *	24.20	75.20	37.00	3.71
HD2967	101.20	11.78	23.40	72.00	36.50	3.63
Mean	103.08	12.07	23.34	72.48	38.5	3.78
SD	4.46	1.34	1.54	5.59	2.52	0.28
CD	3.58	1.18	1.31	7.37	3.8	0.59

PH: Plant Height; SL: Spike length; NSpl: No. of spikelets per spike; NS: No. of seeds per spike; TKW: Thousand Kernel Weight; YLD: Plot yield in kg; * Significantly different from recurrent parent HD2967.

## Data Availability

The data that support the findings of this study are contained within the article.

## Appendix A

**Table A1:** List of 700 markers used in parental polymorphism survey.

S.No.	Marker	S.No.	Marker	S.No.	Marker	S.No.	Marker	S.No.	Marker
1	*Xbarc10*	48	*Xbarc20*	95	*Xbarc95*	142	*Xcfd13*	189	*Xcfd88*
2	*Xbarc101*	49	*Xbarc200*	96	*Xbarc98*	143	*Xcfd143*	190	*Xgdm101*
3	*Xbarc108*	50	*Xbarc206*	97	*Xcfa2019*	144	*Xcfd15*	191	*Xgdm109*
4	*Xbarc109*	51	*Xbarc21*	98	*Xcfa2026*	145	*Xcfd156*	192	*Xgdm113*
5	*Xbarc117*	52	*Xbarc212*	99	*Xcfa2028*	146	*Xcfd16*	193	*Xgdm116*
6	*Xbarc119*	53	*Xbarc229*	100	*Xcfa2037*	147	*Xcfd168*	194	*Xgdm136*
7	*Xbarc121*	54	*Xbarc23*	101	*Xcfa2040*	148	*Xcfd170*	195	*Xgdm14*
8	*Xbarc123*	55	*Xbarc232*	102	*Xcfa2043*	149	*Xcfd190*	196	*Xgdm146*
9	*Xbarc124*	56	*Xbarc24*	103	*Xcfa2056*	150	*Xcfd193*	197	*Xgdm28*
10	*Xbarc127*	57	*Xbarc240*	104	*Xcfa2070*	151	*Xcfd2*	198	*Xgdm33*
11	*Xbarc128*	58	*Xbarc25*	105	*Xcfa2076*	152	*Xcfd2.1*	199	*Xgdm36*
12	*Xbarc13*	59	*Xbarc267*	106	*Xcfa2091*	153	*Xcfd2.2*	200	*Xgdm63*
13	*Xbarc134*	60	*Xbarc28*	107	*Xcfa2104*	154	*Xcfd20*	201	*Xgpw2246*
14	*Xbarc137*	61	*Xbarc3*	108	*Xcfa2106*	155	*Xcfd219*	202	*Xgpw3010*
15	*Xbarc138*	62	*Xbarc32*	109	*Xcfa2110*	156	*Xcfd22*	203	*Xgpw3069*
16	*Xbarc140*	63	*Xbarc37*	110	*Xcfa2114*	157	*Xcfd24*	204	*Xgpw3261*
17	*Xbarc141*	64	*Xbarc4*	111	*Xcfa2121*	158	*Xcfd242*	205	*Xgpw5193*
18	*Xbarc142*	65	*Xbarc40*	112	*Xcfa2123*	159	*Xcfd25*	206	*Xgpw7052*
19	*Xbarc145*	66	*Xbarc417*	113	*Xcfa2129*	160	*Xcfd251*	207	*Xgpw7070*
20	*Xbarc146*	67	*Xbarc45*	114	*Xcfa2134*	161	*Xcfd257*	208	*Xgpw7072*
21	*Xbarc147*	68	*Xbarc48*	115	*Xcfa2141*	162	*Xcfd267*	209	*Xgwm10*
22	*Xbarc148*	69	*Xbarc49*	116	*Xcfa2147*	163	*Xcfd28*	210	*Xgwm107*
23	*Xbarc151*	70	*Xbarc5*	117	*Xcfa2149*	164	*Xcfd283*	211	*Xgwm108*
24	*Xbarc154*	71	*Xbarc55*	118	*Xcfa2155*	165	*Xcfd30*	212	*Xgwm11*
25	*Xbarc158*	72	*Xbarc56*	119	*Xcfa2163*	166	*Xcfd31*	213	*Xgwm112*
26	*Xbarc159*	73	*Xbarc59*	120	*Xcfa2164*	167	*Xcfd36*	214	*Xgwm113*
27	*Xbarc163*	74	*Xbarc60*	121	*Xcfa2170*	168	*Xcfd39*	215	*Xgwm114*
28	*Xbarc164*	75	*Xbarc67*	122	*Xcfa2174*	169	*Xcfd4*	216	*Xgwm120*
29	*Xbarc165*	76	*Xbarc68*	123	*Xcfa2179*	170	*Xcfd48*	217	*Xgwm122*
30	*Xbarc167*	77	*Xbarc69*	124	*Xcfa2183*	171	*Xcfd5*	218	*Xgwm124*
31	*Xbarc17*	78	*Xbarc7*	125	*Xcfa2185*	172	*Xcfd50*	219	*Xgwm126*
32	*Xbarc170*	79	*Xbarc72*	126	*Xcfa2187*	173	*Xcfd54*	220	*Xgwm129*
33	*Xbarc173*	80	*Xbarc73*	127	*Xcfa2190*	174	*Xcfd59*	221	*Xgwm130*
34	*Xbarc174*	81	*Xbarc75*	128	*Xcfa2191*	175	*Xcfd6*	222	*Xgwm131*
35	*Xbarc176*	82	*Xbarc76*	129	*Xcfa2193*	176	*Xcfd60*	223	*Xgwm132*
36	*Xbarc178*	83	*Xbarc77*	130	*Xcfa2219*	177	*Xcfd62*	224	*Xgwm133*
37	*Xbarc18*	84	*Xbarc78*	131	*Xcfa2226*	178	*Xcfd65*	225	*Xgwm135*
38	*Xbarc180*	85	*Xbarc8*	132	*Xcfa2234*	179	*Xcfd7*	226	*Xgwm136*
39	*Xbarc181*	86	*Xbarc80*	133	*Xcfa2240*	180	*Xcfd70*	227	*Xgwm140*
40	*Xbarc182*	87	*Xbarc81*	134	*Xcfa2250*	181	*Xcfd71*	228	*Xgwm146*
41	*Xbarc183*	88	*Xbarc83*	135	*Xcfa2256*	182	*Xcfd73*	229	*Xgwm148*
42	*Xbarc186*	89	*Xbarc84*	136	*Xcfa2257*	183	*Xcfd74*	230	*Xgwm149*
43	*Xbarc187*	90	*Xbarc85*	137	*Xcfa2262*	184	*Xcfd79*	231	*Xgwm153*
44	*Xbarc188*	91	*Xbarc87*	138	*Xcfa2278*	185	*Xcfd80*	232	*Xgwm154*
45	*Xbarc195*	92	*Xbarc89*	139	*Xcfa2293*	186	*Xcfd81*	233	*Xgwm155*
46	*Xbarc197*	93	*Xbarc92*	140	*Xcfd1*	187	*Xcfd82*	234	*Xgwm156*
47	*Xbarc198*	94	*Xbarc94*	141	*Xcfd11*	188	*Xcfd86*	235	*Xgwm159*
236	*Xgwm16*	284	*Xgwm312*	332	*Xgwm471*	380	*Xgwm636*	428	*Xwmc166*
237	*Xgwm160*	285	*Xgwm314*	333	*Xgwm473*	381	*Xgwm637*	429	*Xwmc168*
238	*Xgwm162*	286	*Xgwm319*	334	*Xgwm480*	382	*Xgwm639*	430	*Xwmc169*
239	*Xgwm164*	287	*Xgwm32*	335	*Xgwm493*	383	*Xgwm644*	431	*Xwmc17*
240	*Xgwm165*	288	*Xgwm328*	336	*Xgwm494*	384	*Xgwm66*	432	*Xwmc173*
241	*Xgwm169*	289	*Xgwm33*	337	*Xgwm495*	385	*Xgwm664*	433	*Xwmc175*
242	*Xgwm179*	290	*Xgwm332*	338	*Xgwm497*	386	*Xgwm666*	434	*Xwmc177*
243	*Xgwm18*	291	*Xgwm333*	339	*Xgwm498*	387	*Xgwm666.1*	435	*Xwmc179*
244	*Xgwm181*	292	*Xgwm334*	340	*Xgwm499*	388	*Xgwm666.2*	436	*Xwmc181*
245	*Xgwm182*	293	*Xgwm335*	341	*Xgwm5*	389	*Xgwm67*	437	*Xwmc182*
246	*Xgwm186*	294	*Xgwm339*	342	*Xgwm501*	390	*Xgwm674*	438	*Xwmc183*
247	*Xgwm191*	295	*Xgwm340*	343	*Xgwm508*	391	*Xgwm68*	439	*Xwmc201*
248	*Xgwm192*	296	*Xgwm344*	344	*Xgwm512*	392	*Xgwm70*	440	*Xwmc206*
249	*Xgwm193*	297	*Xgwm350*	345	*Xgwm513*	393	*Xgwm72*	441	*Xwmc213*
250	*Xgwm2*	298	*Xgwm356*	346	*Xgwm515*	394	*Xgwm77*	442	*Xwmc215*
251	*Xgwm205*	299	*Xgwm357*	347	*Xgwm518*	395	*Xgwm88*	443	*Xwmc216*
252	*Xgwm210*	300	*Xgwm359*	348	*Xgwm526*	396	*Xgwm88.1*	444	*Xwmc218*
253	*Xgwm213*	301	*Xgwm361*	349	*Xgwm537*	397	*Xgwm88.2*	445	*Xwmc219*
254	*Xgwm219*	302	*Xgwm368*	350	*Xgwm538*	398	*Xgwm95*	446	*Xwmc230*
255	*Xgwm233*	303	*Xgwm369*	351	*Xgwm540*	399	*Xgwm99*	447	*Xwmc231*
256	*Xgwm234*	304	*Xgwm371*	352	*Xgwm544*	400	*Xwmc1*	448	*Xwmc232*
257	*Xgwm247*	305	*Xgwm372*	353	*Xgwm547*	401	*Xwmc10*	449	*Xwmc235*
258	*Xgwm249*	306	*Xgwm374*	354	*Xgwm55*	402	*Xwmc104*	450	*Xwmc238*
259	*Xgwm251*	307	*Xgwm375*	355	*Xgwm550*	403	*Xwmc105*	451	*Xwmc24*
260	*Xgwm257*	308	*Xgwm376*	356	*Xgwm554*	404	*Xwmc109*	452	*Xwmc243*
261	*Xgwm259*	309	*Xgwm382*	357	*Xgwm558*	405	*Xwmc11*	453	*Xwmc245*
262	*Xgwm260*	310	*Xgwm388*	358	*Xgwm565*	406	*Xwmc110*	454	*Xwmc247*
263	*Xgwm264*	311	*Xgwm389*	359	*Xgwm566*	407	*Xwmc113*	455	*Xwmc25*
264	*Xgwm265*	312	*Xgwm391*	360	*Xgwm569*	408	*Xwmc116*	456	*Xwmc254*
265	*Xgwm268*	313	*Xgwm397*	361	*Xgwm570*	409	*Xwmc118*	457	*Xwmc256*
266	*Xgwm271*	314	*Xgwm4*	362	*Xgwm573*	410	*Xwmc120*	458	*Xwmc257*
267	*Xgwm273*	315	*Xgwm400*	363	*Xgwm577*	411	*Xwmc125*	459	*Xwmc258*
268	*Xgwm274*	316	*Xgwm403*	364	*Xgwm582*	412	*Xwmc128*	460	*Xwmc261*
269	*Xgwm275*	317	*Xgwm408*	365	*Xgwm595*	413	*Xwmc134*	461	*Xwmc262*
270	*Xgwm276*	318	*Xgwm410*	366	*Xgwm6*	414	*Xwmc139*	462	*Xwmc264*
271	*Xgwm282*	319	*Xgwm413*	367	*Xgwm60*	415	*Xwmc145*	463	*Xwmc265*
272	*Xgwm284*	320	*Xgwm415*	368	*Xgwm601*	416	*Xwmc149*	464	*Xwmc269*
273	*Xgwm285*	321	*Xgwm425*	369	*Xgwm604*	417	*Xwmc15*	465	*Xwmc27*
274	*Xgwm291*	322	*Xgwm427*	370	*Xgwm608*	418	*Xwmc150*	466	*Xwmc272*
275	*Xgwm293*	323	*Xgwm429*	371	*Xgwm610*	419	*Xwmc152*	467	*Xwmc273*
276	*Xgwm294*	324	*Xgwm43*	372	*Xgwm611*	420	*Xwmc153*	468	*Xwmc274*
277	*Xgwm296*	325	*Xgwm44*	373	*Xgwm613*	421	*Xwmc154*	469	*Xwmc276*
278	*Xgwm297*	326	*Xgwm443*	374	*Xgwm614*	422	*Xwmc156*	470	*Xwmc278*
279	*Xgwm299*	327	*Xgwm445*	375	*Xgwm617*	423	*Xwmc158*	471	*Xwmc28*
280	*Xgwm30*	328	*Xgwm448*	376	*Xgwm626*	424	*Xwmc16*	472	*Xwmc283*
281	*Xgwm302*	329	*Xgwm459*	377	*Xgwm63*	425	*Xwmc160*	473	*Xwmc289*
282	*Xgwm304*	330	*Xgwm46*	378	*Xgwm630*	426	*Xwmc161*	474	*Xwmc291*
283	*Xgwm311*	331	*Xgwm47*	379	*Xgwm635*	427	*Xwmc163*	475	*Xwmc296*
476	*Xwmc307*	524	*Xwmc453*	572	*Xwmc580*	620	*Xwmc679*	668	*Xwmc776*
477	*Xwmc31*	525	*Xwmc455*	573	*Xwmc581*	621	*Xwmc680*	669	*Xwmc777*
478	*Xwmc310*	526	*Xwmc468*	574	*Xwmc59*	622	*Xwmc682*	670	*Xwmc78*
479	*Xwmc311*	527	*Xwmc469*	575	*Xwmc592*	623	*Xwmc684*	671	*Xwmc783*
480	*Xwmc312*	528	*Xwmc47*	576	*Xwmc593*	624	*Xwmc687*	672	*Xwmc786*
481	*Xwmc313*	529	*Xwmc471*	577	*Xwmc594*	625	*Xwmc692*	673	*Xwmc787*
482	*Xwmc317*	530	*Xwmc473*	578	*Xwmc596*	626	*Xwmc693*	674	*Xwmc79*
483	*Xwmc323*	531	*Xwmc474*	579	*Xwmc597*	627	*Xwmc694*	675	*Xwmc790*
484	*Xwmc326*	532	*Xwmc475*	580	*Xwmc598*	628	*Xwmc695*	676	*Xwmc792*
485	*Xwmc329*	533	*Xwmc476*	581	*Xwmc602*	629	*Xwmc696*	677	*Xwmc794*
486	*Xwmc332*	534	*Xwmc477*	582	*Xwmc603*	630	*Xwmc698*	678	*Xwmc795*
487	*Xwmc335*	535	*Xwmc479*	583	*Xwmc606*	631	*Xwmc70*	679	*Xwmc798*
488	*Xwmc336*	536	*Xwmc48*	584	*Xwmc607*	632	*Xwmc702*	680	*Xwmc805*
489	*Xwmc344*	537	*Xwmc486*	585	*Xwmc611*	633	*Xwmc705*	681	*Xwmc807*
490	*Xwmc349*	538	*Xwmc487*	586	*Xwmc612*	634	*Xwmc707*	682	*Xwmc808*
491	*Xwmc35*	539	*Xwmc488*	587	*Xwmc613*	635	*Xwmc710*	683	*Xwmc809*
492	*Xwmc356*	540	*Xwmc489*	588	*Xwmc615*	636	*Xwmc713*	684	*Xwmc810*
493	*Xwmc361*	541	*Xwmc49*	589	*Xwmc616*	637	*Xwmc716*	685	*Xwmc813*
494	*Xwmc364*	542	*Xwmc491*	590	*Xwmc617*	638	*Xwmc718*	686	*Xwmc815*
495	*Xwmc366*	543	*Xwmc492*	591	*Xwmc619*	639	*Xwmc719*	687	*Xwmc817*
496	*Xwmc376*	544	*Xwmc494*	592	*Xwmc623*	640	*Xwmc722*	688	*Xwmc818*
497	*Xwmc382*	545	*Xwmc497*	593	*Xwmc625*	641	*Xwmc723*	689	*Xwmc819*
498	*Xwmc386*	546	*Xwmc498*	594	*Xwmc626*	642	*Xwmc726*	690	*Xwmc826*
499	*Xwmc388*	547	*Xwmc500*	595	*Xwmc627*	643	*Xwmc727*	691	*Xwmc827*
500	*Xwmc396*	548	*Xwmc505*	596	*Xwmc63*	644	*Xwmc728*	692	*Xwmc83*
501	*Xwmc397*	549	*Xwmc508*	597	*Xwmc630*	645	*Xwmc73*	693	*Xwmc830*
502	*Xwmc398*	550	*Xwmc51*	598	*Xwmc631*	646	*Xwmc734*	694	*Xwmc85*
503	*Xwmc405*	551	*Xwmc511*	599	*Xwmc632*	647	*Xwmc737*	695	*Xwmc89*
504	*Xwmc406*	552	*Xwmc513*	600	*Xwmc633*	648	*Xwmc740*	696	*Xwmc9*
505	*Xwmc407*	553	*Xwmc516*	601	*Xwmc640*	649	*Xwmc744*	697	*Xwmc93*
506	*Xwmc413*	554	*Xwmc517*	602	*Xwmc644*	650	*Xwmc745*	698	*Xwmc95*
507	*Xwmc415*	555	*Xwmc52*	603	*Xwmc646*	651	*Xwmc748*	699	*Xwmc96*
508	*Xwmc416*	556	*Xwmc522*	604	*Xwmc65*	652	*Xwmc75*	700	*Xwmc99*
509	*Xwmc417*	557	*Xwmc524*	605	*Xwmc650*	653	*Xwmc751*		
510	*Xwmc418*	558	*Xwmc525*	606	*Xwmc651*	654	*Xwmc752*		
511	*Xwmc419*	559	*Xwmc526*	607	*Xwmc652*	655	*Xwmc753*		
512	*Xwmc420*	560	*Xwmc527*	608	*Xwmc653*	656	*Xwmc754*		
513	*Xwmc422*	561	*Xwmc532*	609	*Xwmc654*	657	*Xwmc756*		
514	*Xwmc426*	562	*Xwmc533*	610	*Xwmc657*	658	*Xwmc757*		
515	*Xwmc428*	563	*Xwmc537*	611	*Xwmc658*	659	*Xwmc758*		
516	*Xwmc43*	564	*Xwmc539*	612	*Xwmc661*	660	*Xwmc759*		
517	*Xwmc430*	565	*Xwmc540*	613	*Xwmc662*	661	*Xwmc76*		
518	*Xwmc434*	566	*Xwmc544*	614	*Xwmc664*	662	*Xwmc760*		
519	*Xwmc435*	567	*Xwmc546*	615	*Xwmc667*	663	*Xwmc762*		
520	*Xwmc44*	568	*Xwmc553*	616	*Xwmc672*	664	*Xwmc764*		
521	*Xwmc441*	569	*Xwmc557*	617	*Xwmc673*	665	*Xwmc766*		
522	*Xwmc445*	570	*Xwmc559*	618	*Xwmc674*	666	*Xwmc770*		
523	*Xwmc446*	571	*Xwmc577*	619	*Xwmc675*	667	*Xwmc773*		

**Table A2:** List of polymorphic markers used in background selection.

S.No.	Markers	S.No.	Markers	S.No.	Markers	S.No.	Markers
1	*Xbarc10*	22	*Xcfa2170*	43	*Xgwm155*	64	*Xgwm573*
2	*Xbarc128*	23	*Xcfa2187*	44	*Xgwm165*	65	*Xgwm6*
3	*Xbarc148*	24	*Xcfa2193*	45	*Xgwm186*	66	*Xgwm60*
4	*XBarc163*	25	*Xcfa2262*	46	*Xgwm191*	67	*Xgwm613*
5	*Xbarc197*	26	*Xcfd13*	47	*Xgwm192*	68	*Xgwm63*
6	*Xbarc212*	27	*Xcfd193*	48	*Xgwm2*	69	*Xgwm635*
7	*Xbarc229*	28	*Xcfd20*	49	*Xgwm234*	70	*Xgwm66*
8	*Xbarc23*	29	*Xcfd242*	50	*Xgwm249*	71	*Xwmc11*
9	*Xbarc232*	30	*Xcfd39*	51	*Xgwm251*	72	*Xwmc247*
10	*Xbarc417*	31	*Xcfd48*	52	*Xgwm294*	73	*Xwmc291*
11	*Xbarc69*	32	*Xcfd6*	53	*Xgwm304*	74	*Xwmc311*
12	*Xbarc73*	33	*Xcfd71*	54	*Xgwm328*	75	*Xwmc317*
13	*Xbarc83*	34	*Xcfd88*	55	*Xgwm332*	76	*Xwmc417*
14	*Xbarc98*	35	*Xgdm63*	56	*Xgwm334*	77	*Xwmc420*
15	*Xcfa2040*	36	*Xgwm11*	57	*Xgwm350*	78	*Xwmc44*
16	*Xcfa2076*	37	*Xgwm126*	58	*Xgwm382*	79	*Xwmc473*
17	*Xcfa2114*	38	*Xgwm131*	59	*Xgwm403*	80	*Xwmc500*
18	*Xcfa2121*	39	*Xgwm148*	60	*Xgwm46*	81	*Xwmc748*
19	*Xcfa2141*	40	*Xgwm149*	61	*Xgwm493*	82	*Xwmc76*
20	*Xcfa2155*	41	*Xgwm153*	62	*Xgwm495*	83	*Xwmc807*
21	*Xcfa2163*	42	*Xgwm154*	63	*Xgwm513*		
